# Treatment of the Linguistic and Temporal Components of Lexical Activation to Improve Word Retrieval in Aphasia

**DOI:** 10.3389/fresc.2022.824684

**Published:** 2022-02-28

**Authors:** Nadine Martin, Jessica Obermeyer, Julie Schlesinger, Robert W. Wiley

**Affiliations:** ^1^Department of Communication Sciences and Disorders, Temple University, Philadelphia, PA, United States; ^2^Department of Communication Sciences and Disorders, University of North Carolina at Greensboro, Greensboro, NC, United States; ^3^Department of Psychology, University of North Carolina at Greensboro, Greensboro, NC, United States

**Keywords:** impaired lexical activation, verbal short-term memory, temporal processing of words, aphasia treatment, word processing treatment

## Abstract

Current approaches to treatments for word processing impairments in aphasia emphasize two components to target, the linguistic content, semantic or phonological representations of words, and the processing component, access to and retrieval of those representations. In this study, we explore these two components of a treatment to improve lexical activation that supports access and retrieval of word representations. Five people with aphasia participated. The treatment task was repetition of concrete word pairs after a 5-s response delay which was intended to provide practice in maintaining activation of the words for that 5-s period before reproducing them. Two of the five participants demonstrated a difficulty in maintaining activation of single words in repetition, with accuracy decreasing significantly after the 5-s interval. The treatment was applied to all participants, however, to determine if its benefit was specific to those with the activation maintenance impairment. Results confirmed that the activation maintenance treatment in the context of this repetition task led to more treatment gains for the two participants who demonstrated this specific impairment. They made gains on four of the nine measures compared to improvements on one to two measures for the other participants. A second question addressed in this study was the relative importance of the item component (linguistic content) of the treatment and the processing component, maintenance of activation. To that end, there were two conditions of treatment probes, (1) repeated content for all treatment, immediate post-treatment and 3-month maintenance probes and (2) novel content for probes in these three phases of treatment. Only one participant showed significant improvement in treatment when items were novel for all probes. We discuss the possibility that this outcome reflects a more specific deficit in the temporal processing component of lexical activation compared to the two other participants who showed better performance on probes with repeated items in treatment and post-treatment phases. Clinical implications of this study and directions of future research are discussed.

## Introduction

Some current theories of language and aphasia incorporate a central role of short-term memory (STM) in lexical (word) processing, which is realized as short-term maintenance of semantic (meaning) and phonological (sound) representations of words over the course of word retrieval. The interactive activation (IA) model of word retrieval (1) postulates two components of lexical activation that support word processing: rate of activation spread (connection rate) and its maintenance (activation decay rate). Dell and colleagues (2–4) have hypothesized that word retrieval difficulties in aphasia are due to impairment of these activation components. Reduced connection strength slows the speed of activation transmission and the need for more time to access a word's representation. Increased rate of activation decay impairs the short-term maintenance of an activated word representation. The activation impairment can affect primarily transmission or maintenance or a combination of the two parameters. Martin and Dell (5) showed that the nature of impairment is most readily apparent when a response delay is added to a task. In picture naming or word repetition, for example, adding a 5-s response delay will result in three patterns of response compared to a 1-s response delay: increased accuracy (slow transmission benefits from more time to respond), reduced accuracy (poor maintenance leads to too-fast decay of activation), or no change in accuracy (combination of impairments to maintenance and transmission parameters). This account is supported by behavioral and computational studies linking the IA model with picture naming and word repetition data from people with aphasia (3–5). In this study, we use the IA model as a framework for a treatment that targets one of the processing components of word retrieval, activation maintenance. Below, we discuss several features of this model that are relevant to this endeavor.

### Directional Flow of Activation in the IA Model

**Figure 1:**
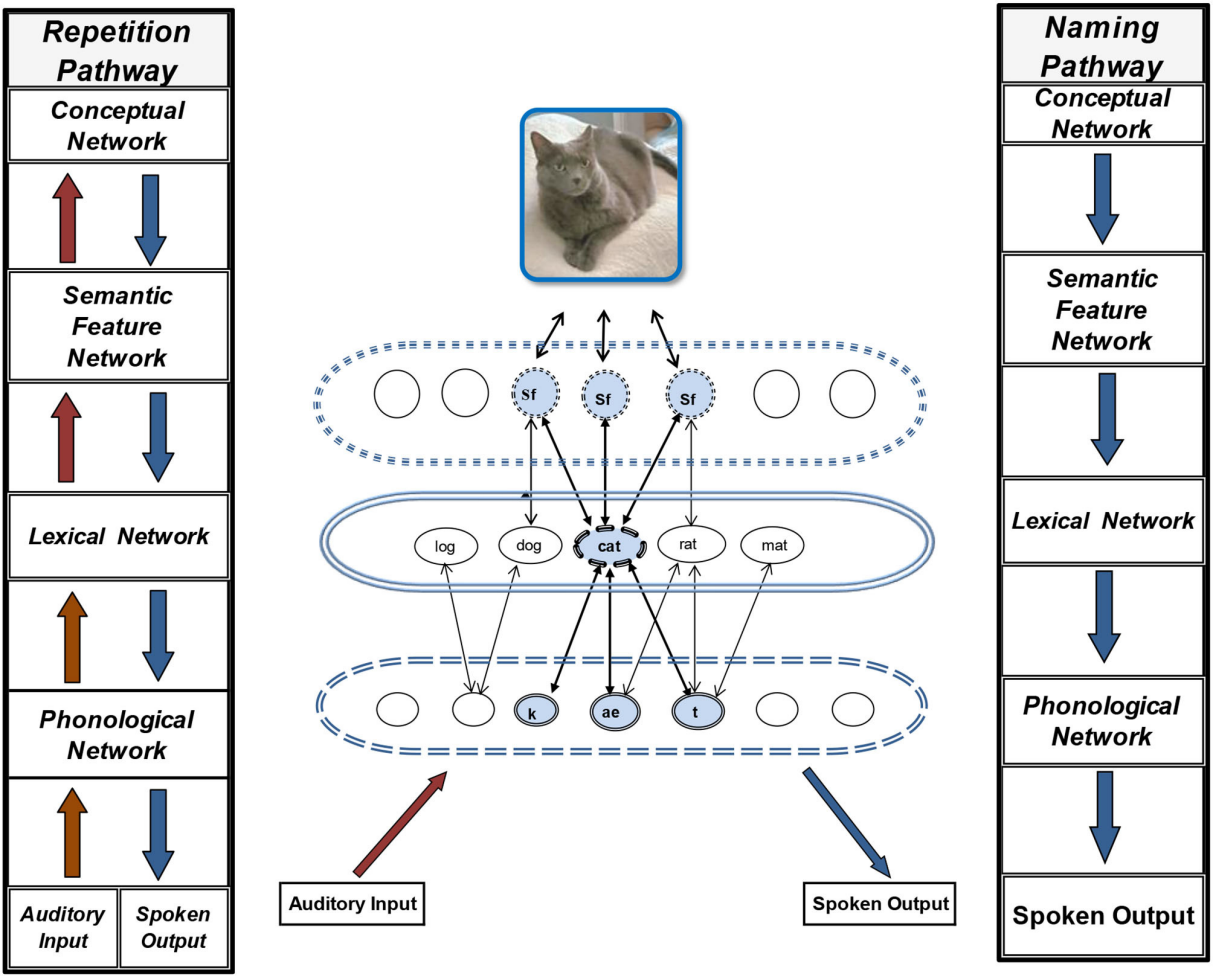
Depiction of the interactive activation semantic-lexical-phonological network for word processing plus two abstracted depictions of the directional flow of activation spread through this network in word repetition and word production.

We have targeted activation maintenance processes in the contexts of repetition [[5]; (6)] and naming tasks (7), both commonly used tasks in treatments of word retrieval disorders in aphasia. Thus, it is useful to consider how the flow of activation across levels of representation in the IA model of word processing differs for these two tasks. Figure 1 shows a depiction of the semantic-lexical-phonological network for word processing in the IA model. Also in this figure are two abstracted depictions of the pathways of activation spread through this network in word repetition and word production (e.g., picture naming). Apart from the overlap of pathways for these two tasks at the output stage (between the lexical and phonological networks), they differ in the type of information that initiates the activation flow and subsequent stages of activation. Repetition begins with auditory input that activates phonological representations of words. Though it gains support through feedforward -feedback activation of lexical and semantic representations as well, it can proceed directly to phonological output stages of production (as in the repetition of non-words). Word production (as in picture naming or self-initiated utterances) follows a path that begins with the concept to be named, moving first through activation of item specific semantic features, which converge on the target lexical (word form) representation, and activate other words to a lesser degree. Activation from each of these activated nodes in the lexicon spreads forward to corresponding sound representations in the phonological network. This activation feeds back to the lexical network, reinforcing word representations that are activated by semantic-lexical activation and activating anew other words that share the sounds activated in the phonological network.

The directional flow of activation is important to designing treatment tasks that target particular connections that are impaired. For example, if input pathways between the lexical and semantic levels of representation are impaired, repetition might not be effective unless it includes stimuli that strongly promote access to semantic representations (e.g., categorically related items). In addition to stimuli considerations, the prominence of activation from one level to another differs depending on the task. In repetition, it is the phonological-to-lexical connections that dominate, while in naming it is the semantic-lexical connections that dominate. The strengths of these connections in relation to levels of impairment (semantic or phonological) should be considered when designing treatment tasks to promote better access to and maintenance of words.

### Why Should We Treat Processing Components of Word Access and Retrieval?

Treating lexical activation processes (e.g., activation maintenance) provides a complement to treatments that target the psycholinguistic content of words (e.g., semantic or phonological). Psycholinguistic approaches add greater precision to treatments compared to early approaches that focused on language abilities (e.g., naming or repetition). And yet, there remains an ongoing challenge of accounting for inconsistent responses to such treatments despite efforts to match the semantic or phonological content to the semantic or phonological impairment [e.g., (8)]. This has been successfully addressed in a recent treatment approach to improve naming that uses two treatment tasks, retrieval practice that addresses semantic-lexical connections and repetition practice that addresses the lexical-phonological connections in naming (9).

In keeping with the theme of this special issue, this study evaluates effects of two components of a treatment that combines lexical priming with activation maintenance. Using a repetition task, the treatment targets the ability to maintain activation of word representations directly, *via* a response delay manipulation and in a second treatment condition, combines this response delay with repetition priming of the words to be repeated. The results suggest that both the linguistic content and processing components of treatment impact the access and retrieval of words for participants, but that these two components may not have equal impact depending on the participant's profile. This will be discussed further in the General Discussion, but we emphasize here, that a deeper understanding of the processes that support access to and retrieval of words (activation transmission vs. maintenance) and their impairment will lead to more refined treatments that target both the linguistic and processing components of language ability and more precise matching of impairment to treatment. Below, we discuss some background empirical studies leading to the current study.

### Treatments Targeting Impairment of Activation Maintenance Processes in Aphasia

The hypothesis that the ability to maintain activation of word representations is impaired in aphasia has motivated clinical applications including diagnostic tools that address effects of increased short-term and working memory load on language processing (10–13) and a growing number of treatments that target short-term maintenance of activation directly (6, 14–17). Here, we report a modified version of a short-term activation maintenance treatment that is embedded in a repetition task, repeating single and multiple word sequences following a response delay. In a previous study (14), we used a single set of items for training, items that would not be trained and probe items. Outcomes of that treatment study were mixed. Repetition improved mostly for the treated stimuli, with limited generalization to untrained items. There was improvement on outcome measures, including single and multiple word processing tasks as well as verbal working memory tasks and verbal spans.

Martin et al. (6) noted that using a single set of items for training, generalization to untrained items and probe items, as Kalinyak-Fliszar et al. (14) did, introduced the potential influence of repeated item exposure on acquisition of trained items. This confound makes it difficult to attribute effects of the treatment exclusively to effects of the verbal short-term maintenance component of the delayed repetition task. To control for the item-exposure variable, Martin et al. (6) used unique items in all phases of the treatment (baseline, training, within and post-treatment probes). The aim was to minimize the effects of repeated exposure and thus isolate the effects of the verbal maintenance treatment on performance of the treatment task as well as generalization to verbal tasks that were similar to the treatment task (near-transfer tasks, e.g., repetition span) and others that were less similar (far-transfer tasks, e.g., picture naming).

Additionally, in the Martin et al. (6) version of this verbal “short-term maintenance” treatment, stimuli were customized for each person based on their performance on screening tasks which involved repetition of concrete and abstract single words, word pairs and word triplets after intervals of 1-s, 5-s, and 10-s. The screener identified the “brink accuracy” of repetition, meaning the combination of variables (stimulus type, sequence length, and interval time, 1-, 5- or 10-s) where performance falters but leaves room for improvement. Based on the results of that screener, we enrolled participants who varied widely in the stimuli used for repetition training (e.g., concrete pairs at a 10-s delay, or abstract word triplets at a 5-s delay). The treatment was designed for individuals who demonstrated an impairment in the short-term activation of words, but Martin et al. (6) included both people with and without this deficit in the sample. This would help to determine whether the treatment's effect was specific to an activation maintenance deficit or was more general. The results suggested that the treatment might be specific to a maintenance impairment: Four of the eight participants who demonstrated an activation maintenance impairment before treatment showed modest acquisition effects coupled with gains in language outcome measures, near transfer tasks that were similar to the training task (e.g., repetition span) and to a lesser degree, far transfer tasks that were less similar to the training task (e.g., naming). The four participants who did not show an activation maintenance impairment before treatment did not improve on the treatment task and showed minimal or no generalization to outcome measures.

The modest acquisition effects in Martin et al. (6) were striking in comparison to those observed in the Kalinyak-Fliszar et al. (14) study using the activation maintenance treatment (repetition + response delay). As discussed above, one important difference between the two studies was that Kalinyak-Fliszar et al. did not control for repeated exposure of trained items. Following the pattern of typical single subject designs, sets of trained and untrained items were chosen and were exposed in probes and in training sessions. The modest acquisition effect when item exposure was minimized suggests that this variable played a role in the outcomes of the Kalinyak-Fliszar et al.'s (14) study. However, the treatment protocol in that study also included a cueing procedure at the start of each training trial in addition to the response delay manipulation. Therefore, we cannot rule out that this contributed to the improvements observed in that study.

### The Present Study

In this study, we sought to elucidate some of the issues surrounding item exposure in treatments to improve short-term maintenance of lexical activation. Following Martin et al. (6), we used novel items for the treatment task but for the probe items, we used a combination of novel and repeated items. To highlight effects of item exposure in this treatment study, we made some adjustments to the treatment stimuli and procedures from earlier studies to bring this variable into focus. Rather than customize the stimuli for the repetition treatment to each individual's repetition and verbal span ability, we limited the stimuli for training to concrete word pairs following a 5-s response delay. This adjustment was intended to simplify the procedures somewhat, since our primary focus was on differences in the acquisition and maintenance of repeated vs. unrepeated (novel) word pairs in the probe task. Additionally, we used concrete words rather than abstract words because of their easier access to semantics (18) and their simpler phonological composition (19). By minimizing potential difficulties in retrieval of the semantic or phonological components of the words, we aimed to minimize this potential confound with effects of repeated exposure of word stimuli. We also aimed to control for span size of each participant so that repeating word pairs would be within their span size and that their span size would not be much >2 words. Concrete word repetition spans ranged from 1.2 to 3 words (details in **Table 3**).

Additionally, we aimed to provide further evidence that this repetition-based treatment to improve short term maintenance of lexical activation will be most effective for those who demonstrate the activation maintenance deficit. As in Martin et al. (6), participants demonstrated different language impairments but also showed different patterns in word repetition accuracy following a response delay. We predicted that those whose repetition was less accurate following a response delay would be most responsive to this treatment.

The following are our research questions for this treatment protocol that uses uniquely exposed words as training stimuli in a delayed word pair repetition paradigm:

1. Will effect sizes for word pairs that are repeated across all probes be greater than effect sizes for word pairs that are unique in each probe?
2. Will performance on outcome measures improve after this treatment?
3. Will improvements on outcome measures be most robust for those participants who show a maintenance deficit in repetition?

In a *post-hoc* analysis, we review accuracy scores of the participants on selected subtests of the Temple Assessment of Language and Short-term memory in Aphasia [TALSA; (10)] that assess the effects of time interval on performance, including naming, repetition of words and non-words. The tasks that include a time interval between stimulus and response are similar to the training task and therefore might indicate some pattern of performance that is consistent with the participants' responses to the treatment.

## Methods and Materials

### Research Design

We used a single subject treatment design including the following phases: baseline assessment, treatment, post-treatment assessment, and a 3-month post-treatment follow-up.

### Participants

#### Biographical Information

Five participants with aphasia were enrolled in the treatment study after meeting criteria on a screener. All five participants were male and at least 1 year post-onset a left hemisphere stroke. Ages ranged from 50 to 61 (Mean = 55, SD = 4.18), time post onset ranged from 45 to 192 months (Mean = 107.4, SD = 57.84), and education level ranged from 7 to 17 years (Mean = 12.4, SD = 3.91). WAB-R (20) Aphasia Quotients ranged from 66.3 to 78.7 (Mean = 74.36, SD = 4.96). Biographical information for these participants is presented in Table 1 and includes the individual Aphasia Quotients.

**Table 1:** Biographical information.

**ID**	**Sex**	**Age**	**MPO^a^**	**Education in years**	**Etiology**	**WAB-R^b^ aphasia quotient**	**WAB-R aphasia classification**
CN39	M	53	63	10	Left MCA^c^ CVA^d^	76.3	Transcortical Motor
KC3	M	57	192	14	Left MCA CVA	77.4	Transcortical Sensory
KG62	M	54	111	14	Left MCA CVA	66.3	Broca's
KK55	M	61	126	17	Left MCA CVA	78.7	Anomic
XH46	M	50	45	7	Left MCA CVA	73.1	Conduction

a *MPO, months post-onset*.

b *WAB-R, Western Aphasia Battery-Revised (20)*.

c *MCA, middle cerebral artery*.

d *CVA, cerebral vascular accident*.

Subjects voluntarily enrolled in this study by signing a consent form approved by the Institutional Review Board at Temple University. All testing and treatment took place from 2017 to 2018 at the Eleanor M. Saffran Center for Cognitive Neuroscience at Temple University.

### Screening Procedures

#### Evidence of a Repetition Impairment

To identify people that would be appropriate for this study, we adapted one of the Auditory Processing subtests from the Psycholinguistic Assessments of Language Processing in Aphasia [PALPA, (21)] to create a repetition screener. Stimuli for the screener were a mix of 1-, 2-, and 3-syllable words arranged so that each string included two words, was four syllables in length and all strings were balanced for low or high frequency. Scores were determined by string accuracy and then a percentage was derived. Anyone with a score of 80% accuracy or greater on the word pairs after a 5-s delay was considered at ceiling and did not continue with the treatment. To ensure a participant was able to complete the task of repetition of word pairs, they were required to get at least one pair correct to move forward with the treatment (see Supplementary Figure 1).

#### Word Processing Abilities With Response Delay and Memory Load Manipulations

Table 2 shows performance on five subtests from the Temple Assessment of Language and Short-term memory in Aphasia [TALSA; (10)] including picture naming, word and non-word repetition and two working memory tests involving judgment of synonymy and rhyming. Details of the stimuli can be found in Martin et al. (10). We will focus first on the word and non-word repetition subtests that will determine whether participants demonstrated an activation maintenance impairment in repetition. Recall that worse performance after a delay signals difficulty in maintaining activation long enough to achieve or sustain access to semantic and phonological representations of a word. Better performance on a task after a response delay indicates that activation is slow to rise and a time delay benefits performance. The treatment task is repetition of word pairs after a 5-s response delay. Two participants, KK55 and XH46, show a decline in repetition accuracy after a 5-s interval for both words and non-words. The other participants, CN39, KC3, and KG62, show similar accuracy rates on the 1- and 5-s delay conditions or in one case, greater accuracy on the 5-s condition. By this measure, KK55 and XH46 represent the repetition profile that is well-suited to this treatment using repetition after a response delay. If treatment gains are limited to these two individuals, this will provide additional evidence that the activation maintenance treatment is most effective when applied to individuals who demonstrate the activation maintenance impairment.

**Table 2:** Effects of response delay and memory load word processing: proportion correct by participants on subtests of the Temple Assessment of Language and Short-term Memory in Aphasia (TALSA).

**TALSA subtest**										
**Participant**	**Naming**		**Word repetition**		**Non-word repetition**		**Rhyming triplets**		**Synonymy triplets**	
**Participant ID**	**1-s delay**	**5-s delay**	**1-s delay**	**5-s delay**	**1-s delay**	**5-s delay**	**Low memory load**	**High memory load**	**Low memory load**	**High memory load**
CN39	0.71	0.77	0.91	0.91	0.38	0.47	0.97	0.90	0.90	0.90
KC3	0.72	0.79	0.89	0.89	0.24	0.29	0.87	0.70	0.83	0.58
KG62	0.50	0.59	0.56	0.67	0.13	0.11	0.90	0.77	0.80	0.68
KK55	0.81	0.82	0.98	0.84	0.53	0.27	0.93	0.73	0.90	0.83
XH46	0.43	0.56	0.91	0.71	0.56	0.38	0.83	0.70	0.70	0.60

There are a few other noteworthy observations regarding the performances of KK55 and XH46 (Table 2). KK55's performance on all of the naming, repetition and working memory tests is higher or amongst the highest of the group at the 1-s interval. It is at 5-s that his performance falters. In naming, XH46 improves after 5 s, a hallmark of an activation transmission deficit. This suggests that his word processing deficit includes both activation maintenance and transmission components, with the latter impacting naming more than repetition. Consistent with this he achieved higher scores on tests that tap into phonological ability (repetition and rhyming judgments) compared to those that probe semantic abilities (naming and synonymy judgments). Finally, the Rhyming Triplets and Synonymy Triplets investigate the ability to judge similarity of meaning and sound under high and low working memory conditions. All five participants' scores decline in the high working memory load condition (with one exception, CN39 on the synonymy triplets). This is a common pattern on these two judgment tasks for people with aphasia and to a lesser extent, neurotypicals (22). KK55, one of the two participants who demonstrated the activation maintenance deficit profile for repetition, scored at a high level for both rhyming and synonymy triplets in the low memory load condition. However, in the high memory load condition, his performance declines considerably and more so for the rhyming triplets, which tap into phonological processing. XH46's performance on these two judgment tasks is lower than KK55 and at the low end for all participants in both memory load conditions and in both rhyming and synonymy triplets.

### Stimuli Development

Stimuli for all baselines, probes, post-treatment probes, and other lab-developed assessments discussed further in this paper were derived from Brysbaert et al. “Concreteness ratings for 40 thousand generally known English word lemmas” (23). We selected only nouns and further reduced the list to include only 1-, 2-, and 3-syllable words. We excluded homophones and other words that the research team felt were inappropriate for our purposes (e.g., “slang” words). To identify concrete and abstract words, we set a criterion of 0.75 standard deviation from the mean of concreteness ratings. Words with ratings of 4.03–5 were considered concrete and words with ratings 1.44–2.77 were considered abstract. We used ~1,900 concrete and 200 abstract 1–4 syllable words.

#### Baselines and Probes

All words were further controlled for frequency using SUBTLEX_WF_ (Subtitle frequency: word form frequency) (24) with ratings limited to between 1 and 25 per million. Once we identified a corpus of words, we developed baselines and probes. The following criteria were set: repeated pairs remained consistent across each probe. The words in a pair could not be semantically related or strongly associated. Additionally, the words in a pair could not share the same initial phoneme, final phoneme, or stressed vowel. We attempted to follow the criteria for phonological similarity as closely as possible for all probes.

We also controlled as much as possible other shared features of words within a probe list, such as balancing for number of animals or food items in each probe and considered phonological features as well by attempting to balance for words ending with /o/, /r/, and /l/ within a single probe. All words were then controlled for phonotactic probability. For baselines, probes and post-treatment probes, concrete word pairs were always 5- or 6-syllables in total, with the 5-syllable strings being combinations of 2- + 3-syllable and 3- + 2-syllable words. For each probe list of 20 pairs, 15 of the strings were repeated and five strings were novel for each of the three (or four) baselines, eight probes, three immediate post-treatment probes, and two maintenance probes for a total of 16 probes throughout the treatment.

#### Treatment

All words chosen for the treatment lists were not used in any of the other lab-developed tests. Since the words developed for the treatment were not used for scoring purposes, criteria for word choice were less strict. SUBTLEX_WF_ ratings for treatment stimuli did not have an upper limit. There was a wider range of frequencies that allowed for anything >25. To increase the number of concrete 3-syllable words, we included some compound words and some pseudo-repetition (kayak/kayaker, balloon/balloonist).

For treatment lists, all concrete word pairs were made of 5- and 6-syllable strings, with the 5-syllable strings balanced in 2- + 3-syllable and 3- + 2-syllable word combinations. Words were not semantically related within a line. It was more difficult to control for shared initial or final consonant or stressed vowel in each line, but since these were treatment lists, this was not considered to be as essential as with the probe lists.

### Pre- and Post-treatment Assessments and Outcome Measures

The language and short-term memory assessments described below were administered immediately before treatment, immediately after treatment and 3 months following the completion of treatment to assess maintenance (except for the discourse task which is only reported for pre- and post-treatment). These assessments are described below.

#### Concrete and Abstract Word and Word Sequences Repetition Test

This laboratory-developed assessment (6) was used to evaluate improvement of word repetition ability. This version included eight repetition conditions that varied the number (pairs, triplets) and concreteness (concrete/abstract) of words and the response delay time (1-s or 5-s) resulting in eight combinations: Concrete Pairs 1-s, Concrete Pairs 5-s, Concrete Triplets 1-s, Concrete Triplets 5-s, Abstract Pairs 1-s, Abstract Pairs 5-s, Abstract Triplets 1-s, and Abstract Triplets 5-s.

For each condition, we administered 20 pairs of words (used only in that condition) that were balanced for syllable length within the pair or triplet and within the condition. Pairs were made up of five syllable strings that were balanced into 2- + 3-syllable combinations and 3- + 2-syllable combinations. Triplets were made of 7- and 8-syllable strings. The 7-syllable strings were balanced into combinations of 2- +2- +3-syllables, 2- + 3- + 2-syllables, or 3- + 2- + 2-syllables. Finally, the 8-syllable strings were balanced into combinations of 2- + 3- +3-syllables, 3- + 2- + 3-syllables or 3- + 3- + 2-syllables. Administration of these forms was pseudo-randomized so that duplicated conditions were not given on the same day (e.g., concrete pairs with a 1-s response delay was not given on the same day as concrete pairs after a 5-s response delay). All stimuli criteria listed above for lab-developed tests also applied in the development of these test forms.

#### Concrete Immediate Serial Recall Span Test

This laboratory-developed test was adapted from the span test we used in the first version of this treatment study (6), using most of the same words but in rearranged order. For this version, we had word strings ranging from one to six words with 10 trials for each list length. Words within a string appeared only once. All stimuli criteria listed previously were also used for developing this test.

#### Word Pointing Span

Each participant received a Word Pointing Span task that was developed as part of the Temple Assessment of Language and Short-term Memory in Aphasia (TALSA) (10). This was included to determine if the treatment improved the ability to maintain activation of verbal representations. Using the pointing span paradigm allows assessment of this ability in the context of a comprehension (word-to-picture-matching) task, without a verbal response.

#### Corsi Block Span Task

We administered this spatial span task as a measure of non-verbal span (25, 26). If the effect of our treatment is on verbal processing and short-term memory only, there should be little or no change in non-verbal span.

#### Comprehensive Aphasia Test (CAT)

The following subtests of the CAT (27) were administered: Comprehension of Spoken Language (Spoken Words, Spoken Sentences, and Spoken Paragraphs) as well as the Naming Objects subtest under Spoken Language Production.

#### Discourse

We administered the Nicholas and Brookshire (N&B) (28) elicitation protocol as this is shown to be a reliable pre-/post-measure of discourse.

### Protocol

Testing was administered by three individuals; a licensed clinical and research speech-language pathologist and two post-doctoral fellows with a Ph.D. in speech and language pathology, one of whom also was a licensed clinical speech-language pathologist. The treatment schedule was prepared a priori on a calendar to ensure administration uniformity among testers. One of the research speech-language pathologists who was involved in administering the first version of this treatment (JS) provided training to the other two testers (JO, IM) before the start of administration.

#### Pre-treatment Assessment Battery

All language tests listed above were given over 5–6 sessions concurrent to administering baselines.

#### Probes

Baseline probe administration began during the pre-treatment assessment battery. Probe trials consisted of 20 word pairs of concrete nouns, 15 repeated across probe trials and five pairs that were unique to each probe trial. On a probe trial, the participant listened to a word pair and when cued after 5 s, repeated the word pair in the order that it was presented. The probe task was administered at the beginning of each session. At least three baseline probes were administered with an optional 4th baseline probe for any participant who demonstrated a change of >15 percentage points on any of the first three administrations. During the treatment phase, probes were administered at the start of eight out of nine treatment sessions, with the exception of the first session, in which no probe was administered. There were also three immediate post-treatment probes (these probes occurred within 1 to 2 weeks following the completion of treatment) and two maintenance probes administered 3 months after treatment.

#### Control Task

A linguistic and non-linguistic control task was administered following each probe during baseline, treatment, post-treatment, and maintenance phases. The linguistic control was the 24-item non-word reading list from the PALPA (21). Because this treatment is intended to promote a verbal-STM process that is fundamental to all language tasks, it was difficult to choose a linguistic control task that we would expect to not improve following this treatment. However, we also expect that the benefits of this treatment will vary depending on the degree of overlap between processes and representations engaged in the training task (repetition with a response delay) and those engaged in a task targeted for generalization. Generalization of positive effects to other tasks can be classified as near transfer or far transfer (6, 29). The non-word reading task would be considered a far transfer task relative to the repetition training task, though we submit that this does not preclude the possibility of performance on this task improving following this treatment.

For a non-linguistic control we used the Five-Point Test (5PT) (30). This test requires participants to generate designs using different combinations of dots and lines. We used this measure to test the hypothesis that improved performance on the outcome measures could be attributed to improved maintenance of lexical activation and not to a more domain general cognitive processing ability.

#### Timing of Response Delays and Periodic Rest Breaks

E-Prime 2.0 software (31) was used to present stimuli electronically to facilitate the clinician's monitoring of the timing of stimulus presentation rate (words within sequences) and the timing of response delays (1-s and 5-s). Natural transitions served as breaks between each task. In addition, breaks were offered if clinical judgment determined it was needed or the participant requested a break.

#### Treatment

Treatment took place over nine sessions, three sessions per week for 3 weeks. The treatment protocol was repetition of 40-word concrete pairs after a 5-s delay. Participants listened to a word pair and waited for a beep cue, which occurred when 5 s had passed. They would then repeat the words in the same order they were presented. Timing and beep cues were programmed into E-prime for accuracy. Treatment stimuli consisted of balanced 2- and 3-syllable words in pairs all of which were novel.

Each session began with a 20-item list probe of concrete pairs (15 repeated strings and 5 novel) followed by a linguistic and non-linguistic control task, the order of which was alternated each session. An example of a probe form is included in the Supplementary Materials.

Treatment began after the treatment probe and two control probes were administered. The treatment and probe tasks were the same: the participant listened to a concrete word pair, waited 5-s until a cue to respond, and then repeated the word pair as accurately as possible. Again, an E-Prime program was used to control for the clinician's presentation rate of the word pairs and the 5-s response delay condition. Each day's treatment was broken into two cycles. Each cycle consisted of two sets—Set A and Set B. Each set consisted of 10 pairs.

### Scoring

#### Accuracy of Word Production

The criteria for accuracy of word production in all probes and all outcome measures was 100% phoneme accuracy. We accepted distortions of phoneme production as long as the phoneme was recognizable. We also accepted regionalisms. For example, it is common in the Philadelphia area to pronounce “ambulance” as /æmb?læns/ so this was considered correct.

#### Scoring of the Probe Tasks

Sessions were audio recorded and following each session, the examiner listened to the sound file to score the responses. Four scores were calculated from the word pair probe data, all expressed as percentages correct:

1. Strings correct in serial order (String ISO). This occurred when the participant produced both words in the pair correctly and in the same order they were presented. The score for each trial could be 0 or 1 out of 1.
2. Strings correct in any order (String IAO). This occurred when the participant produced both words in the pair without regard to order. Score for each trial could be 0 or 1 out of 1.
3. Words correct in serial order (Words ISO). This was a measure of the total items in the word pair produced correctly. Score for each trial could be 0, 1, or 2 out of 2.
4. Words correct in any order (Words IAO). This was a measure of the total items in the word pair produced correctly without regard to order. Score for each trial could be 0, 1, or 2 out of 2.

So, if the target is *recipe, arcade* and the response after the beep is “recipe, arcade,” that word pair would be scored as 1 out of 1 for String ISO/String IAO and 2 out of 2 for total Words ISO/IAO. In another example, if the target is *thunder, coconut* and the response after the beep is “thunder, /kod
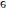
k
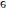
n
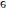
t/, coconut” that would be considered 0 out of 1 for String ISO as it was not completely correct but would receive a 1 out of 1 for string IAO, 1 out of 2 for total words ISO and 2 out of 2 for total words IAO. See Supplementary Materials for a probe that is filled in using this scoring method.

### Reliability

For the probe task, reliability was evaluated by having a trained undergraduate volunteer serve as a second scorer. For probes, each participant had one baseline, one immediate post-test, and one maintenance probe randomly selected for rescoring, which was ~17.65% of the total amount of probes administered to each person. Substantial agreement was seen for strings ISO (kappa = 0.688) scoring for probe responses (32).

### Statistical Analyses

To address Research Question 1, we calculated effect sizes across word pair types and time points using linear mixed-effects regression. Specifically, binomial regression was used to regress accuracy on the two predictors of interest as well as their interaction. The regression models were fit in R with the package *lme4* [version 1.1-26; (33)]. A separate model was fit to each individual participant's data, with fixed effects for Time Point (Baseline, Immediate post-treatment, 3 months post-treatment, simple coded with Baseline as the reference level), Word Pair Type (Repeated and Novel, coded +1/-1), the interaction between Time Point and Word Pair Type, and a random intercept by-items. Accuracy on the two control tasks (PALPA Non-words and 5-Point Test) were analyzed in the same way, except the regression was fixed-effects only, with the only regressor being Time Point (and so the models were fit in the base R package *stats* [v. 4.0.5, (34)]. Effect sizes are all reported as odds ratios (OR).

To address Research Question 2, the various outcome measures were either scored and compared against established benchmarks, as described in the Results, or else were analyzed with regression similar to the approach described for Research Question 1. Specifically, the laboratory-developed Concrete and Abstract Word and Word Sequences Repetition Test was analyzed with (binomial) fixed effects regression, with regressors for Time Point, Delay (1-s vs. 5-s), String Size (Pairs vs. Triplets), and Word Type (Concrete vs. Abstract; each of these categorical variables had their levels coded as +1/-1. The interactions between Time Point and each of the other regressors were also included. Therefore, a single regression model (per participant) provided estimates and *p*-values for the “main effect” of Time Point, as well as whether that effect differed under the various conditions. For example, the interaction of Time Point X Delay tested whether any changes from pre-treatment to immediate-post treatment (or from pre- treatment to 3-months post-treatment) were different for words tested at 1-s vs. 5-s. Only significant interactions with Time Point were followed-up with comparisons to report condition-specific changes in accuracy across time (e.g., a significant interaction of Time Point X Delay was followed up with a test of Time Point at 1-s delay and Time Point at 5-s delay), using the R package emmeans [v. 1.5.5-1; (35)].

Discourse was evaluated pre-treatment and post-treatment. The Discourse elicitation protocol described by Nicholas and Brookshire (28) was used to evaluate the connected speech of each participant. All 10 samples from the Nicholas and Brookshire (28) protocol were used and included single picture descriptions (4), sequential picture descriptions (2), procedural discourse samples (2), and personal narratives (2). Results of the 10 samples were totaled for each participant. The primary discourse outcome was the proportion of correct information units (CIUs). CIUs are words that are accurate and relevant to the stimuli and not repeated (28). The proportion of CIUs was calculated by totaling the number of CIUs across all 10 discourse samples divided by the total number of words produced in all 10 discourse samples for each participant. Additionally, number of words, number of CIUs and mazes (false starts and filled pauses in discourse) were evaluated for each participant. Discourse transcription was completed by trained research assistants. Point-to-point reliability was evaluated for 17% of transcripts with 93.5% agreement. Transcription reliability was determined by dividing total agreed upon words, utterances, false starts, filled pauses, and silent pauses (pauses >2 s) over the total number possible. Point-to-point coding reliability was also evaluated for 48% of transcripts. Agreement for words was 99.4% (total agreed upon words over total words) and agreement for CIUs was 89.9% (total agreed upon CIUs over total CIUs). The primary discourse outcome (%CIUs) was evaluated using the benchmark of change greater than twice the standard error of the mean (4.2%) established by Nicholas and Brookshire (28) and used to evaluate change in %CIUs after treatment (36, 37).

To evaluate Research Question 3, we examined the performances of all five participants on the treatment probes as well as outcome measures, to determine whether those participants who demonstrated a maintenance deficit in repetition benefited from the treatment more so than those who did not.

## Results

### Research Question 1

**Figure 2:**
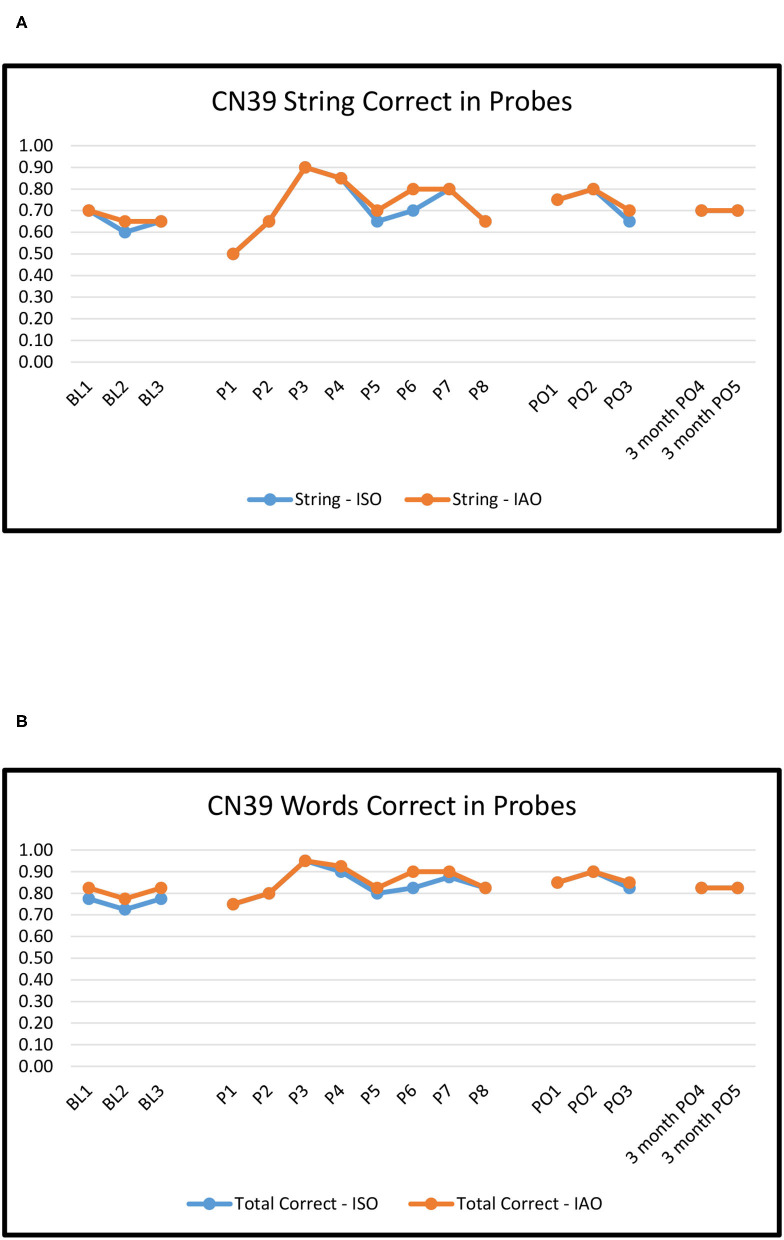
CN39: Proportion of word strings **(A)** and words **(B)** correct in baseline, treatment, post-treatment, and 3 months follow-up probes.

**Figure 3:**
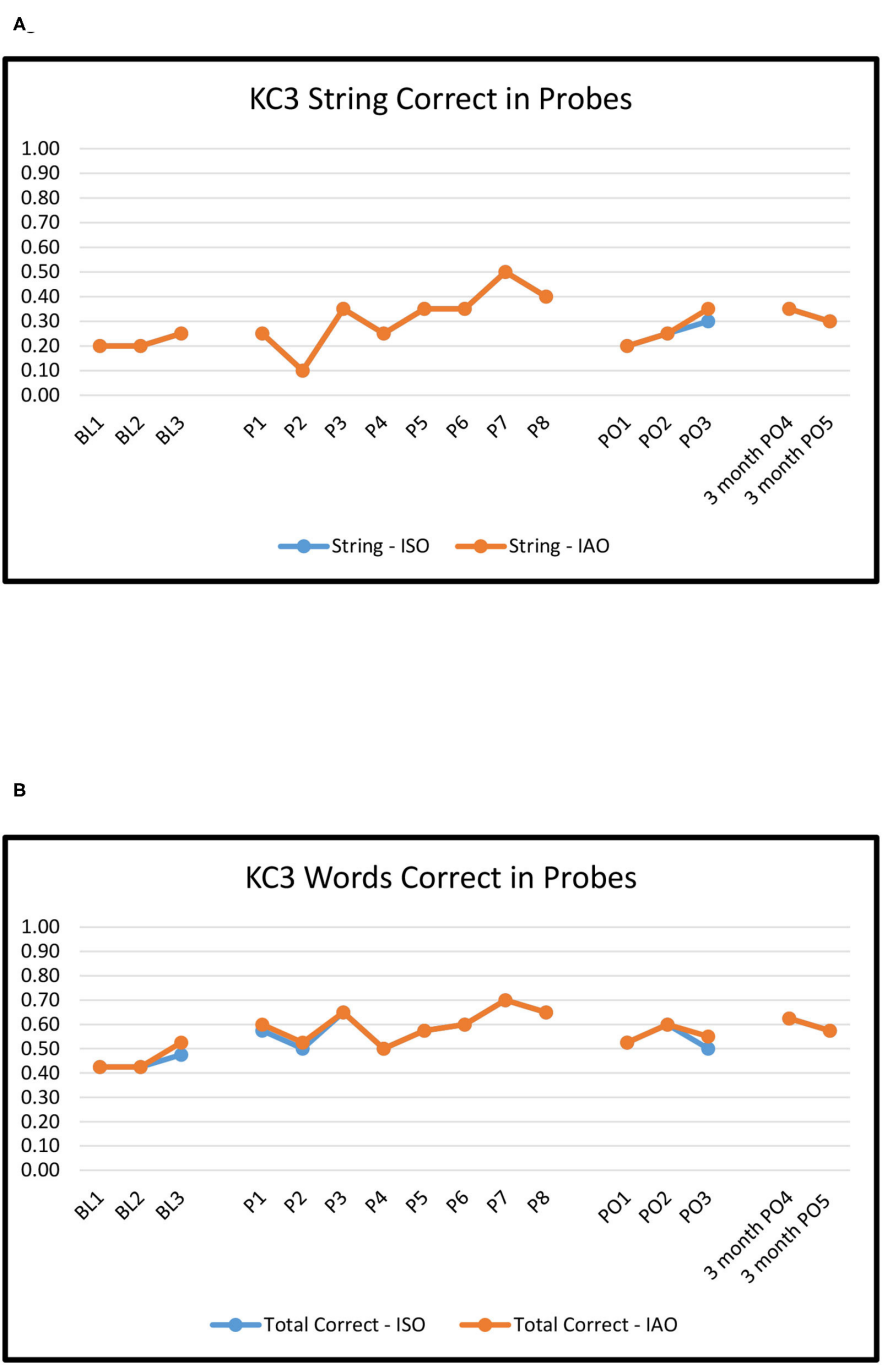
KC3: Proportion of word strings **(A)** and words **(B)** correct in baseline, treatment, post-treatment, and 3 months follow-up probes.

**Figure 4:**
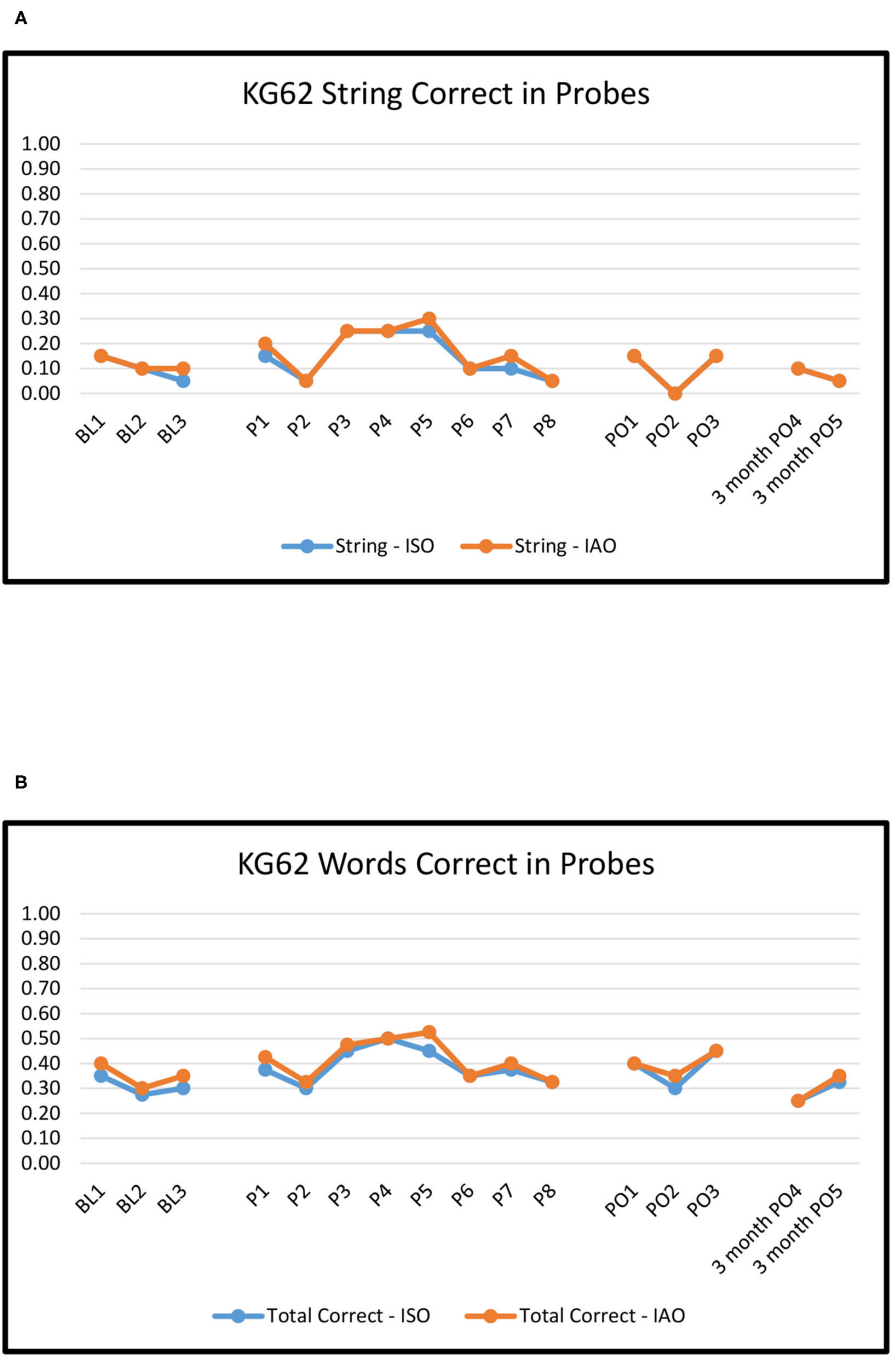
KG62: Proportion of word strings **(A)** and words **(B)** correct in baseline, treatment, post-treatment, and 3 months follow-up probes.

**Figure 5:**
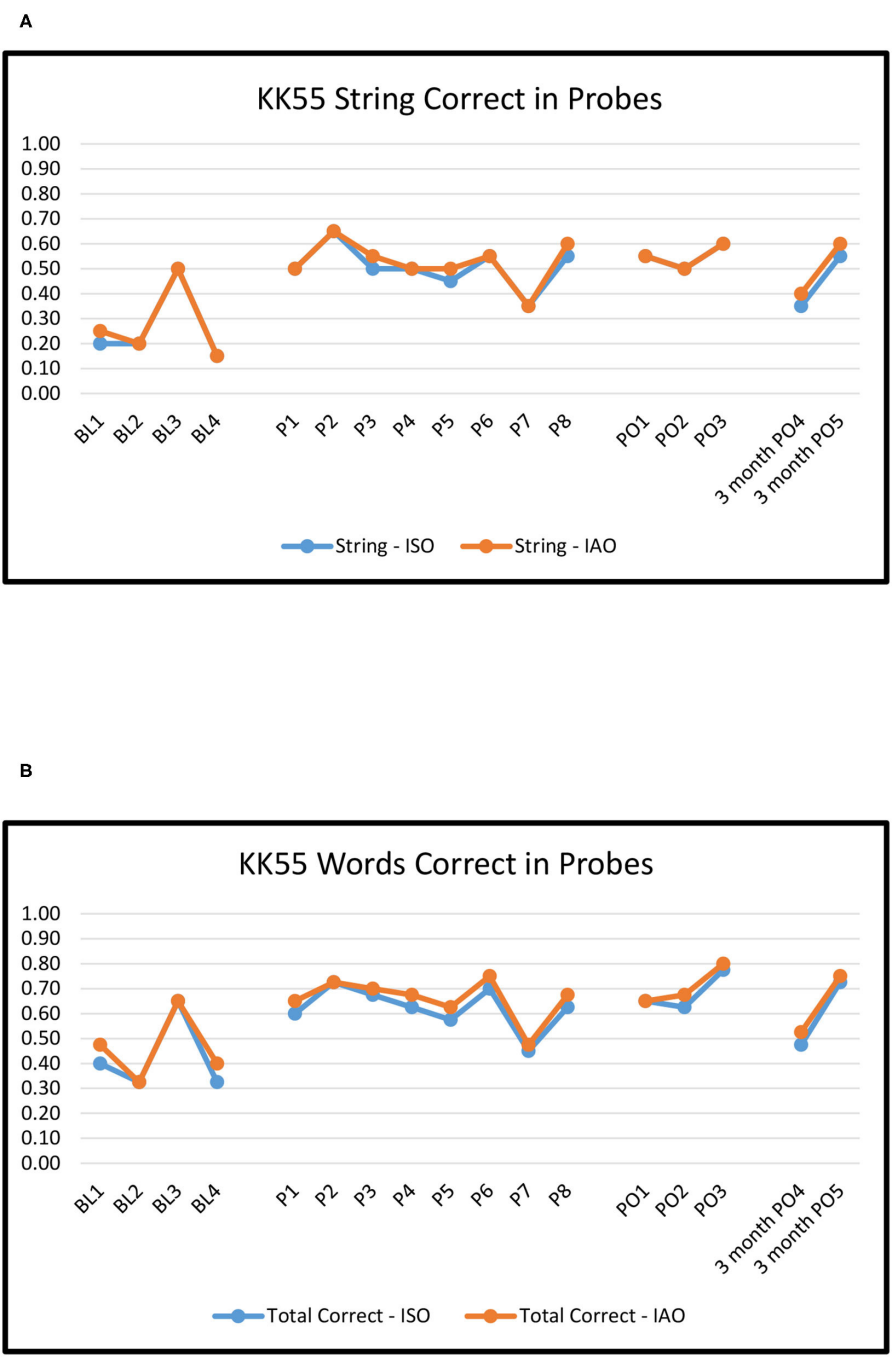
KK55: Proportion of word strings **(A)** and words **(B)** correct in baseline, treatment, post-treatment, and 3 months follow-up probes.

**Figure 6:**
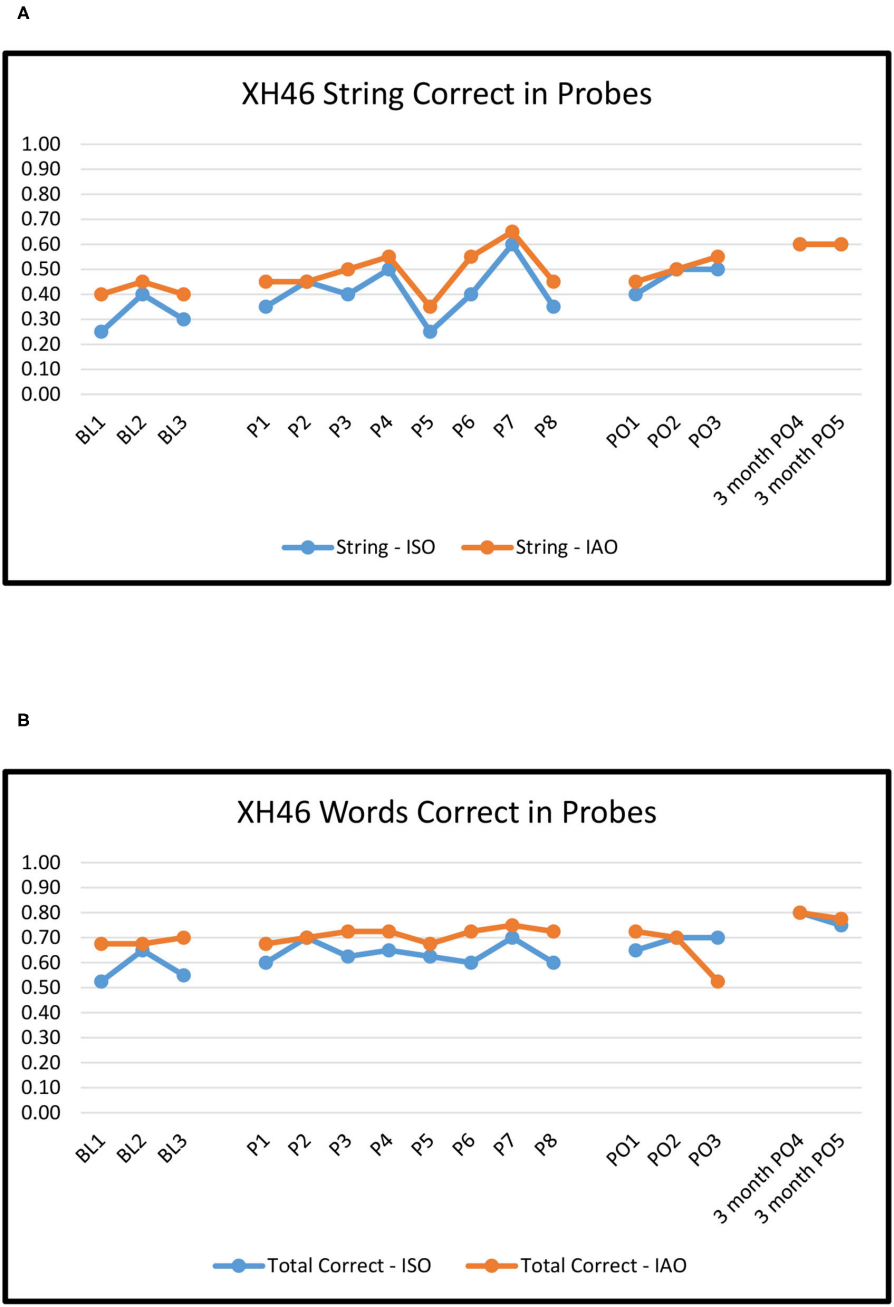
KK55: Proportion of word strings **(A)** and words **(B)** correct in baseline, treatment, post-treatment, and 3 months follow-up probes.

Figures 2–6 show the results of baseline, treatment, post-treatment, and follow-up probe trials for each participant. The results are expressed as proportions of strings correct ISO and IAO (2a-6a) and proportions of words correct ISO and IAO (2b-6b).

The first research question asked whether treatment effect sizes for word pairs repeated across all probes would be greater than effect sizes for word pairs that are unique in each probe? “Overall” effects refer to collapsing across pair Type (Repeated and Novel). An odds ratio (OR) < 1 indicates a decrease in accuracy, whereas an OR > 1 indicates improvement. An OR equal to exact 1 indicates no change whatsoever. Strings ISO refers to the proportion of word strings recalled accurately in serial order. Words ISO refers to the proportion of words recalled within strings and in serial order.

#### Effect Sizes for Changes on Probes From Pre-treatment to Immediate Post-treatment and to 3 Months Post-treatment

Summaries of the results for each participant on the treatment measures are provided below.

#### CN39

For baseline to immediate post-treatment, across the two outcome measures (Strings ISO, Words ISO), Overall ORs ranged from 1.6 to 2.2, canonical small effects (38). Of those, only Words ISO (OR = 2.20) was marginally significant (*p* ≈ 0.06); all other effects were not significant, *p*'s > 0.10. The ORs for Novel pairs, ranging from 1.8 to 2.7, were numerically larger than those for Repeated pairs, ranging from 1.3 to 1.8. However, there were no significant differences between Novel and Repeated pairs, *p*'s > 0.10.

For baseline to maintenance (3 months post-treatment), Overall ORs ranged from 1.3 to 2.1. None of the effects, however, were significant (*p*'s > 0.10). The ORs for Novel pairs, ranging from 1.6 to 5.1, were numerically larger than those for Repeated pairs, ranging from 0.8 to 1.4. However, there were no significant differences between Novel and Repeated pairs, *p*'s > 0.10.

#### KC3

For baseline to immediate post-treatment, Overall ORs ranged from 0.5 to 1.1, none of which were significant (*p*'s > 0.10). However, large and significant differences emerged between Novel and Repeated pairs. The ORs for Novel pairs ranged from 0.04 to 0.5, with String ISO (OR = 0.04) showing a marginally significant *decrease* in the odds of a correct response (*p* ≈ 0.06). The ORs for Repeated pairs ranged from 2.4 to 6.9. Results for Repeated pairs indicated significant improvements with a medium effect for Words ISO and a large effect for String ISO. The differences between Novel and Repeated pairs were significant for both of these outcome measures (*p*'s < 0.05).

For baseline to maintenance, the pattern was largely the same, with non-significant *decreases* in accuracy on Novel pairs (ORs 0.07–0.5, *p*'s > 0.10), but significant maintenance of improvement on Repeated pairs with large effect sizes (ORs 3.5–17.4, *p*'s < 0.01). The differences between Novel and Repeated pairs were significant for Strings ISO and Words ISO.

#### KG62

For baseline to immediate post-treatment, overall ORs ranged from 0.8 to 1.6, none of which were significant (*p*'s > 0.10). The effects were numerically more positive for Novel pairs (ranging from 1.0 to 2.2) than Repeated pairs (ranging from 0.6 to 1.2). However, none of the effects or differences between Novel and Repeated pairs were significant (*p*'s > 0.10).

For baseline to maintenance, the pattern was largely the same: no significant changes on either Novel or Repeated pairs (ORs 0.5–1.0, *p*'s > 0.10) and no significant differences between the two.

#### KK55

For baseline to immediate post-treatment, Overall ORs ranged from 3.2 to 3.9, medium effects, all of which were significant (*p*'s < 0.01). The effects were of very similar magnitude for Novel pairs (ranging from 2.8 to 4.0) and Repeated pairs (ranging from 3.2 to 3.9), with no significant differences between the two (*p*'s > 0.10).

For baseline to maintenance, Overall ORs ranged from 1.3 to 1.9, small effects, none of which were significant (*p*'s > 0.10). The effects were numerically larger for Novel pairs (ranging from 1.2 to 2.9) than for Repeated pairs (ranging from 1.2 to 1.7), however none of the effects were significant nor were they different from each other (*p*'s > 0.10).

#### XH46

For baseline to immediate post-treatment, overall ORs ranged from 1.3 to 1.6, none of which were significant (*p*'s > 0.10). Effects for Novel pairs ranged from 0.7 to 1.1, none of which were significant (*p*'s > 0.10). However, effects for Repeated pairs ranged from 1.5 to 3.5, with significant improvement for Strings ISO and Words ISO (*p*'s < 0.01). The difference between Novel and Repeated pairs was marginally significant for String ISO (*p* ≈ 0.8), driven by significant improvement for Repeated pairs but (non-significant) declines for Novel pairs.

For baseline to maintenance, overall ORs ranged from 1.2 to 2.2, none of which were significant (*p*'s > 0.10). Effects for Novel pairs showed non-significant declines (ORs 0.6–0.7), whereas effects for Repeated pairs (ORs 2.4–6.6) showed improvements that were significant for Strings ISO and Words ISO (*p*'s < 0.05). The difference between Novel and Repeated pairs for Words ISO was marginally significant (*p* ≈ 0.06), driven by significant improvement on Repeated pairs and (non-significant) declines on Novel pairs.

#### Summary

Participants CN39 and KG62 showed no significant changes from baseline, at either immediate post-treatment or maintenance.

KC3 showed significant improvement on Repeated pairs, with large effect sizes for Strings ISO (ORs 6.7 and above) and small-to-medium effects for Words ISO. KC3's improvements on Repeated items were significantly greater than on Novel pairs (which showed no significant changes). These improvements were maintained 3 months after finishing treatment.

KK55 showed significant improvements on both Novel and Repeated items immediately post-treatment, with medium effect sizes (ORs 2.8–3.9). However, improvements were not significantly maintained 3 months after treatment.

Finally, XH46 showed significant improvement only for Repeated items, for Strings ISO or Words ISO; the effect sizes were small-to-medium immediately post-treatment (ORs 2.3–3.5) but were medium-to-large 3 months after treatment (ORs 3.6–6.6).

### Research Question 2

Will this treatment that combines repeated and novel stimuli in the probe stimuli lead to improvements in outcome measures? Results of an analysis of outcome measures before and after treatment and at 3 months post-treatment are reported below.

#### Concrete and Abstract Word and Word Sequences Repetition Test

No significant changes were observed on the String ISO outcome measure; the following results all reflect changes in Words ISO.

#### CN39

There was a significant interaction between Time Point (baseline vs. 3 months) and String Size (OR = 0.73, *p* < 0.05). Follow-up comparisons revealed that this interaction was driven by a significant decrease from baseline to 3 months post-treatment for pairs (65 to 53%; OR = 0.60, *p* < 0.05) contrasting with a trend toward an increase for triplets (41 to 43%; OR = 1.09, *p* ≈ 0.64).

#### KC3

There was a marginal interaction between Time Point (baseline vs. 3 months) and String Size (OR = 0.76, *p* ≈ 0.08). Follow-up comparisons revealed a similar pattern of results as observed for CN: the interaction was driven by a marginally significant increase for triplets (19 to 26%; OR = 1.47, *p* ≈ 0.08) contrasting with a trend toward a decrease in accuracy for pairs (46 to 42%; OR = 0.84, *p* ≈ 0.43).

#### KG62

There was a significant main effect of Time Point (baseline vs. immediate post-treatment), reflecting an overall improvement from 12 to 16% (OR = 1.74, *p* < 0.05). There was also a significant interaction between Time Point (baseline vs. 3 months) and Word Type (OR = 1.78, *p* < 0.05). Follow-up comparisons revealed the interaction was driven by a marginally significant increase for abstract words (4 to 7%, OR = 2.25, *p* ≈ 0.11) contrasting with a trend toward a decrease for concrete words (21 to 16%, OR = 0.73, *p* ≈ 0.24).

#### KK55

There was a marginal interaction between Time Point (baseline vs. 3 months) and String Size (OR = 1.41, *p* ≈ 0.07). Follow-up comparisons revealed the interaction was driven by a marginally significant decrease in accuracy for triplets (15 to 9%; OR = 0.59, *p* ≈ 0.07) contrasting with a trend toward an increase in accuracy for pairs (31 to 35%; OR = 1.18, *p* ≈ 0.48).

#### XH46

There was a significant interaction between Time Point (baseline vs. 3 months) and Delay (OR = 1.49, *p* < 0.01), and marginally significant interactions between Time Point (baseline vs. immediate post-treatment) and Duration (OR = 1.29, *p* ≈ 0.09) and Time Point (baseline vs. 3 months) and Word Type (OR = 0.77, *p* ≈ 0.09). Because of the presence of interactions between Time Point and both Delay and Word Type, we tested for the presence of a 3-way interaction; this was found to be significant, OR = 1.32, *p* ≈ 0.014. Follow-up comparisons revealed the interaction was driven by significant decreases particular to abstract words tested at the 5-s delay, contrasting with no change or improvements at 1-s and for concrete words. The specific pattern was: at 1-s delay, there were no significant changes for abstract words (*p*'s > 0.10), but at 5-s, abstract words decreased significantly from baseline to immediately post-treatment (44 to 27%, OR = 0.45, *p* < 0.05) and remained near significantly below baseline at 3 months (28%, OR = 0.55, *p* ≈ 0.053). This contrasts with concrete words, which showed no effects of duration but rather numerical improvement from baseline to immediate post-treatment (38–39%) and marginally significant improvement from baseline to 3 months (38 to 47%, OR = 1.42, *p* ≈ 0.08).

#### Summary

In terms of improvements on this task, one participant (KG62) showed significant gains overall, while two individuals (KC3 and XH46) showed marginally significant gains specific to certain conditions. Specifically, KC3 marginally improved on Triplets at 3 months post-treatment, and XH46 marginally improved on Concrete words at 3 months post-treatment. KG62, who improved significantly in general from baseline to immediate post-treatment, remained marginally significantly better at Abstract words 3 months post-treatment. Neither CN39 nor KK55 showed any improvements (*p*'s > 0.10); instead, they showed some declines (CN39 performed significantly worse on Pairs at 3 months post-treatment; KK55 performed marginally worse on Triplets 3 months post-treatment).

Verbal and non-verbal span tasks. The results of three span measures are reported in Table 3.

**Table 3:** Spans in serial order (ISO) and in any order (IAO) at pre-treatment, immediate post-treatment, and 3 months maintenance.

	**Participant ID**										
		**CN39**		**KC3**		**KG62**		**KK55**		**XH46**	
**Span task**	**Time Point**	**ISO**	**IAO**	**ISO**	**IAO**	**ISO**	**IAO**	**ISO**	**IAO**	**ISO**	**IAO**
Concrete word repetition span	Pre-tx	3	3	2	2	1.2	1.2	2.2	2.2	2	2.8
	Immediate post-tx	3	3	2	2	1.8	1.8	2	2	2.4	2.6
	3 mo post-tx	3.4	3.4	2	2	1.2	1.2	2.2	2.2	3.0^*^	3.2
Word pointing span	Pre-tx	4.2	4.2	2	2.2	3	3	2.4	2.6	2.4	3
	Immediate post-tx	3.8	3.8	2	2	2.6	2.8	2.6	2.8	3	3
	3 mo post-tx	4.4	4.4	2	2.2	3.4	3.4	2.4	2.4	3	3
Corsi block span	Pre-tx	5.7	6	5	5.3	4.7	7	4	4	5	6
	Immediate post-tx	5.7	6.7	4.3	5.7	4.7	5.7	4	4.7	5	5.3
	3 mo post-tx	5.3	6	4	4.7	5.3	5.7	4.3	4.3	4.7	6

* *Increase of 1.0 in span is considered to be noteworthy improvement*.

#### Concrete Immediate Serial Recall Span Test

Using Log Odds ratios, we looked at pre- and post-spans to determine improvement. One person, XH, showed an improvement from 2.0 ISO span before treatment to a span of 3.0 ISO at maintenance (trend: *p* = 0.0655). As a participant in the version of this treatment reported by Martin et al. (6), XH46's performance on the Concrete Immediate Serial Recall Span Test improved from 1.4 ISO pre-treatment to 2.4 ISO post-treatment. Thus, his concrete word span, as measured in this study, shows continued improvement.

#### Word Pointing Span Task

No significant gains in word pointing span were observed for any participant from baseline to immediate post-treatment and maintenance testing at 3 months.

#### Corsi Block Span Task

No significant gains were observed on this measure of non-verbal span for any participant from baseline to immediate post-treatment and maintenance testing at 3 months.

#### Comprehensive Aphasia Test (CAT)

Results of the Comprehensive Aphasia Test (27) are shown in Table 4. All subtests of the Comprehension of Spoken Language (Spoken Words, Spoken Sentences, and Spoken Paragraphs) as well as the Naming Objects subtest under Spoken Language Production were compared pre-treatment and post-treatment using t-scores to determine change. Benchmarks varied for significant improvement and were taken from the CAT manual. Two of the five participants showed some significant improvement. For Comprehension of Spoken Language, KK55 went from a t-score of 43 at baseline, to 57 at immediate post-treatment, and 55 at maintenance. XH46 improved in Spoken Language Production (naming objects) with a *t*-score of 51 at baseline to 59 at maintenance. These results are shown in Table 4. It is worth noting here that XH46 also showed improvement in naming [on the Philadelphia Naming Test, (39)] in the version of this therapy reported by Martin et al. (6).

**Table 4:** Comprehensive Aphasia Test (CAT) subtest T-scores at pre-treatment (pre-tx), immediate post-treatment (imm post), and 3 months maintenance (3 mo post).

		**CN39**			**KC3**			**KG62**			**KK55**			**XH46**		
**Language domain**	**Subtest**	**Pre-tx**	**Imm post**	**3 mo post**	**Pre-tx**	**Imm post**	**3 mo post**	**Pre-tx**	**Imm post**	**3 mo post**	**Pre-tx**	**Imm post**	**3 mo post**	**Pre-tx**	**Imm post**	**3 mo post**
	Spoken words	58	53	58	49	45	55	60	51	58	46	53	53	58	65	65
Comprehension of spoken language	Spoken sentences	65	60	57	41	43	44	53	52	54	43	57	55^*^	65	67	65
	Spoken paragraphs	49	49	43	43	49	43	49	49	49	49	43	49	60	60	60
	Total score	61	56	55	43	43	46	53	50	53	43	53^*^	52^*^	62	67	65
Spoken language production	Naming objects	64	60	61	53	54	54	53	50	50	62	66	61	51	52	59^*^

Significant T-score changes (indicated by an ^*^) vary by subtest and are taken from Table 4.3 in the CAT manual. For Spoken Words and Spoken Sentences the change is 9, for Spoken Paragraphs the change is 10, for Total Comprehension of Spoken Language the change is 7, and for Naming Objects the change is 7.

#### Discourse Samples

One of the five participants demonstrated evidence of improvement on the primary discourse outcome (%CIUs), while one participant demonstrated a decline. At pre-treatment, KK55 produced 49% CIUs which increased to 60% at post-treatment. His total number of words at pre-treatment were 670 and 486 at post-treatment. Total number of CIUs produced were 327 at pre-treatment and 290 at post-treatment. This combination of higher %CIUs and lower total words indicates an increase in his efficiency of relevant content production. CN39 produced a smaller proportion of mazes when comparing pre-treatment to post-treatment performance. At pre-treatment he produced 22% mazes which were determined by dividing the total number of mazes (false starts and filled pauses) over the total words produced. At post-treatment, %mazes decreased to 14% which could indicate improved efficiency of lexical retrieval since false starts and filled pauses are often considered behavioral indicators of lexical retrieval difficulty (40). KG62 demonstrated a decline in %CIUs when comparing pre-treatment and post-treatment performance. At pre-treatment he produced 50% CIUs and at post-treatment 40% CIUs. Total words (pre-treatment = 291, post-treatment = 324) and total CIUs (pre-treatment = 146, post-treatment = 130) were consistent with this decline and indicated a reduction in efficiency and relevant content which was evidenced by the production of more words and fewer CIUs.

#### Performance on Control Tasks Before and After Treatment and at 3 Months Post-treatment

Individual performance on the control tasks is detailed below.

#### CN39

The PALPA non-word reading accuracy increased significantly from 11% at baseline to 26% immediately post-treatment, OR = 2.87, *p* < 0.05. At 3 months post, the score remained marginally significant above baseline at 23%, OR = 2.38, *p* ≈ 0.09. The 5-point drawing test (5PT) decreased significantly from 61% at baseline to 39% immediately post-treatment, OR = 0.42, *p* < 0.01, but there was no significant difference between baseline and 3 months post-treatment (53%, OR = 0.75, *p* ≈ 0.39).

#### KC3

The PALPA score increased significantly from 5% at baseline to 20% immediately post-treatment, OR = 4.10, *p* < 0.05. At 3 months post, the score remained significantly above baseline at 19%, OR = 3.92, *p* < 0.05. The 5PT increased significantly from 68% at baseline to 85% immediately post-treatment, OR = 2.67, *p* < 0.05, but there was no significant difference between baseline and 3 months post-treatment (78%, OR = 1.75, *p* ≈ 0.23).

#### KG62

The PALPA score showed no significant changes, neither from baseline (5%) to immediately post-treatment (4%, OR = 0.74, *p* ≈ 0.70), nor to 3 months post (6%, OR = 1.13, *p* ≈ 0.87). The 5PT decreased significantly from 43% at baseline to 23% immediately post-treatment, OR = 0.38, *p* < 0.01, and remained significantly below baseline to 3 months post-treatment (24%, OR = 0.41, *p* < 0.01).

#### KK55

The PALPA score increased significantly from 25% at baseline to 53% immediately post-treatment, OR = 3.35, *p* < 0.001. At 3 months post, the score remained significantly above baseline at 46%, OR = 2.54, *p* < 0.5. The 5PT showed no significant changes from baseline to immediate post-treatment (unchanged at 61%, OR = 0.98, *p* ≈ 0.96) or 3 months post-treatment (57%, OR = 0.82, *p* ≈ 0.60).

#### XH46

The PALPA score showed no significant changes, neither from baseline (0%) to immediately post-treatment (0%) nor to 3 months post (0%). The 5PT likewise showed no significant changes, from 37% at baseline to 31% immediately post-treatment (OR = 0.78, *p* ≈ 0.50), and 38% at 3 months post-treatment (OR = 1.06, *p* ≈ 0.88).

#### Summary of Performance on Control Tasks

##### Linguistic Control, Non-word Reading

CN39, KC3, and KK55 showed significant improvements immediately post-treatment and this improvement was maintained at 3 months (significant for KC3 and KK55 and marginally significant for CN39).

##### Non-linguistic Control, the Five-Point Test

KC3 increased significantly immediately following treatment, but this was not maintained at follow-up. Other participants did not show any significant improvement on this test immediately following treatment or at follow-up.

### Research Question 3

Will improvements in outcome measures be most robust for those participants who show a maintenance deficit in repetition?

Two participants, KK55 and XH46, demonstrated the activation maintenance impairment with accuracy of word and non-word repetition declining after a 5-s response delay (Table 2). KK55 demonstrated significant improvement on the treatment task for both repeated and unrepeated items from baseline to post-treatment and baseline to maintenance (3 months post treatment). XH46 showed significant improvement for repeated items at post-treatment and at maintenance. Of those who did not show the maintenance impairment in repetition, only KC3 showed improvement in word repetition after this treatment for repeated items immediately post-treatment and at 3 months maintenance.

On outcome measures, KK55 showed significant improvement on the comprehension of spoken sentences subtests of the CAT and XH46 improved significantly on the naming subtest of the CAT. On the span tasks, XH46's span for concrete words increased from 2 to 3. On Discourse measures, KK55 improved significantly on the rate of CIUs produced. On the repetition of concrete-abstract sequences test, KG62 showed an overall main effect from baseline to immediate post-test, and XH46 showed a significant decrease in accuracy specific to abstract words at a 5-s delay.

In summary, three participants showed significant effects sizes for the treatment, KK55, XH46, and KC3, but only KK55 showed these effects for repeated and novel probes. KK55 and XH46, who demonstrated the activation maintenance deficit in repetition, also made gains on outcome measures as detailed above.

These findings indicate that this treatment is most effective with individuals who show an activation maintenance deficit in repetition, KK55 and XH46. To illustrate the improvement by the two participants relative to other participants in this treatment study, Table 5 shows nine language and verbal span measures where evidence can be found for improvement. KK55 and XH46 made gains on four of the nine measures, followed by KC3 who improved on two and then CN39 and KG62 who each improved on one of the nine measures.

**Table 5:** Summary of gains made on the outcome measures.

	**Measure**	**CN39**	**KC3**	**KG32**	**KK55**	**XH46**
1	Treatment effect sizes - repeated probes		**+**		**+**	**+**
2	Treatment effect sizes - novel probes				**+**	
3	Concrete and abstract word sequence repetition		**+** ^*^	**+**		**+** ^*^
4	Concrete words immediate serial recall					**+**
5	Word Pointing span					
6	CAT Spoken Language Comprehension				**+**	
7	CAT Spoken Word production (Naming)					**+**
8	Discourse: Increased CIUs				**+**	
9	Discourse: Decreased % Mazes	**+**				
	Total of measures showing some improvement	**1**	**2**	**1**	**4**	**4**

***+** signifies an effect significant at p < 0.05*.

+^*^ signifies a marginally significant effect.

## Discussion

The treatment described here is a follow-up from the treatment reported by Martin et al. (6) in which item exposure in a word sequence repetition treatment was minimized to reveal effects of a 5-s response delay, which invokes short-term maintenance of activated word representations. By tackling the difficulty in maintaining activation of representations directly, we aimed to improve this ability that supports access to and retrieval of words in repetition, naming and other language tasks. The results of that study were different from our prior studies [e.g., (14, 41)] that combined the response delay manipulation with a set of treated, untreated and probe items used in all phases of treatment (baseline through maintenance). In this study, we investigated more closely the effect of response delay with and without the added influence of repeated item exposure. As in the Martin et al. (6) study, some participants improved following this treatment while others did not. Some possible reasons for this outcome are offered below.

Starting with our initial aim, we first wanted to know if an effect of repeated exposure would be evident for items repeated in the probe trials compared to those items that were unique on each probe trial. Two participants, CN39 and KG62, showed no improvement on repeated or novel probes. For those individuals who benefited from the treatment, KC3 and XH46 showed significant improvement on the repeated probe items compared to the novel probe items. KK55 showed comparable levels of improvement on both repeated and novel probe item conditions.

In Martin et al. (6), effects of the maintenance treatment with minimal repetition of stimuli were modest overall, but still, improvements in outcome measures were observed for four of the eight participants. A similar pattern was observed in this study with two of the participants who demonstrated the activation maintenance impairment showing gains in several outcome measures. KK55 improved on the CAT sentence comprehension test and the primary discourse measure, % CIUs. XH46 improved in naming on the CAT test and showed a span increase from 2 to 3. This increase demonstrates continued improvement from the change in his span that was observed when he participated in the Martin et al. (6) study (span increased from 1.4 to 2.4).

XH46's continued gains in span abilities in this second round of a version of the activation maintenance treatment raises the question of effectiveness of multiple treatments distributed over time. KC3 and CN39, whose gains were more limited, also participated in the Martin et al. (6) activation maintenance treatment study. Although evidence favors the benefit of multiple treatment periods distributed over time [e.g., (42)], the span of time between participation in these two studies ranged from 15 to 24 months. With this amount of time and the likelihood of participation in numerous communication activities in the interim, we considered this to be a new treatment for these three individuals rather than a continuation of their previous participation in a similar treatment. Nonetheless, it is worth noting that XH46 showed continued improvement in this second round of the activation maintenance treatment.

We also observed changes in the two control tasks following therapy, especially the linguistic control, non-word reading. Three participants improved on this measure, CN39, KC3, and KK55. In hindsight, this outcome is not completely surprising given the nature of the treatment—practice in maintaining access to words (their activation) sufficiently for longer periods of time. This ability is fundamental to language processing, and improvements in this ability could result in improvements on other language tasks besides the treatment task. Non-word reading is considered a distant measure, meaning there is not much overlap with the repetition task used for treatment. However, non-words are potential words and reading does share output production processes with repetition. Thus, some extension of improvements to this task may be expected. Of greater concern is the improvement seen for participant KC3 on the 5-point test, the non-linguistic control task. However, this improvement was observed in the immediate post-treatment testing but was not sustained in the 3 months maintenance testing period. This finding could also be indicative of the potency of repeated trials in acquisition, similar to the repeated probe items.

### Understanding the Linguistic and Processing Components of This Treatment and Word Processing Impairments in Aphasia

The activation maintenance treatment combines repetition with a response delay. Here, we disentangled influences of the linguistic component (the words to be repeated) and the activation maintenance component (the response delay) by varying the exposure of items in the probes, with some repeated across all probe trials and others novel across probe trials. The results indicate that the treatment was successful for some but not all the participants, and more successful when items in probes were repeated. As we develop and refine this treatment approach toward its eventual use in the clinic, it is worth addressing a few questions about the approach and its potential as a clinical tool.

#### Why Do Some People Respond to This Treatment and Not Others?

One of the most important issues to be addressed in aphasia rehabilitation is why some people with aphasia respond well to an impairment-based treatment while others do not. An obvious first thought is that the treatment does not match up with the impairment. With broad diagnoses such as Broca's aphasia or fluent aphasia, it is likely that there will be enough variability in symptoms within a diagnostic category that some people with that diagnosis would not respond well to a treatment designed for its cardinal symptoms. A related concern is that other cognitive abilities (e.g., attention) may be impaired and are somehow compounding the language impairment. As diagnostic tools become more detailed in their descriptions of an impairment, the matches between treatment and impairment type should fit more closely. Psycholinguistic models and linguistic theory, for example, have guided development of tests that probe access to linguistic elements of words and sentences [e.g., (21, 43)], providing more precise measurements of impairments to language function.

The activation maintenance treatment is an outgrowth of another variable of impairment to language ability, the processing component. Our knowledge of the components of activation processes that support language is increasing [e.g., (1, 44, 45)]. Studies also have revealed how impairment to processing components impacts language performance (2–5, 46). Martin and Dell (5) provide evidence for two processing parameters, activation transmission and activation maintenance, that regulate access and retrieval of words. This study provides further evidence that the activation maintenance component of language processing is a viable treatment target for certain participants. Similar to Martin et al. (6), we found that participants with poor maintenance of activated word representations in repetition, KK55 and XH46, made the most gains on the treatment task and outcome measures (see Table 5).

#### What Is the Nature of the Separate and Combined Linguistic and Activation Maintenance Components of This Treatment?

KK55 was the only participant who improved on both novel and repeated probe items. Further, KK55 demonstrated improvement on several outcome measures including the CAT and the primary discourse outcome (%CIUs). At pre-treatment, KK55's scores on word and non-word repetition were higher than the other participants in the 1-s condition (see Table 2) and his scores dropped after 5 s. Thus, KK55's pre-treatment profile suggests that access to linguistic information (the activation transmission component of language processing) is less problematic for him than maintaining access to those representations. XH46 also presents with a maintenance deficit, but when comparing his pre-treatment assessment results and his response to this treatment, the pattern is quite different than KK55. XH46's language performance is more impaired than KK55's in the 1-s condition of the repetition and two working memory tasks, synonymy and rhyming triplets, and it becomes even more impaired in the 5-second condition.

Additionally, XH46's accuracy on the TALSA naming subtests (Table 2) improves after a 5-s response delay, which is the signature of an activation transmission deficit. How can we account for the XH46's task specific activation impairments, transmission in naming and maintenance in repetition? We suggest that XH46's lexical activation impairment includes both transmission and maintenance components and that the manifestation of these deficits differs depending on the task and the locus of impairment. As described in the Introduction, in word production, activation spreads from activated semantic representations to an arbitrarily related word form. In repetition, activation spreads from an input sequence of phonemes to a phonological word form. It is conceivable that the spread of activation from semantics to the lexical form is more vulnerable to a transmission impairment than the input phonological activation to a phonological word form. Regarding the locus of impairment, XH46's performance on all subtests reported in Table 2 was impaired, but it was less accurate on those subtests with a substantial semantic component (naming and synonymy triplets). In treatment, XH46's improvement on the repeated probe items suggests that this condition provided priming of the semantic-lexical representations needed to facilitate the transmission between these levels of representation. A broader message of this finding is that both lexical priming (i.e., repeated exposure of training items) and processing (activation transmission or maintenance) treatments may be needed for more severely impaired language abilities or when there are different severity levels of impairment to semantic vs. phonological processes.

#### How Do the Activation Maintenance and Transmission Treatments Fit With Current Taxonomies of Treatment Approaches?

Recent developments in rehabilitation science provide a framework for evaluating principles and components of treatment approaches, the Rehabilitation Treatment Specification System [RTSS, (47, 48)], that can be applied to various rehabilitation practices (e.g., physical therapy, occupational therapy). Turkstra et al. (49) propose the application of this system to practice in speech and language pathology and in a recent series of papers (50–53), a group of researchers in aphasia rehabilitation considered the value of the RTSS framework for evaluating rehabilitation approaches in aphasia. RTSS evaluates three aspects of a treatment: the target (behavior that the treatment will change), the treatment ingredients (essential elements of the treatment) and the mechanism(s) of action (how a treatment works). Within this framework, the characterization of the treatment reported here and its variants (6, 14) could be the following: The target is improved access to words in the context of various language tasks and the endurance of that activation. There are three ingredients in this treatment: the task (repetition, but could be another language task, e.g., naming), a response delay and repetition priming *via* repeated exposure of probes and/or training items (6). Martin et al. (10) demonstrated adverse or beneficial effects of a response delay on performance of many language tasks, allowing for flexibility in the choice of therapy target and task. Logically speaking, the response delay should be essential to a treatment that aims to improve maintenance of activation, simply because it targets the deficit directly. It could also be a sufficient ingredient for some [e.g., KK55 in this study and four of the participants in the Martin et al. (6) study]. For others, though, lexical priming may be needed in combination with a response delay to improve performance after a 5-s response delay. It is not certain whether lexical priming alone (through repeated practice on probe items in this study) could be sufficient to improve the ability to maintain activation of a word to the extent that repetition accuracy increases after a response delay. This possibility would be difficult to test because the definition of an activation maintenance impairment is accurate repetition with no delay in response and impaired repetition after a response delay. The evidence thus far suggests that lexical priming in combination with a response delay is effective for some participants and for others, targeting the response delay alone (with the novel lexical items) improves accuracy after the delay, suggesting improvement in activation maintenance ability. Further studies are needed to learn how to detect impairments that involve each component of repetition—lexical activation and maintenance of that activation, or some combination of these. As we investigate variations of this paradigm in future studies, we note that RTSS characterization of task components has served as a useful starting point to understanding the cognitive-linguistic mechanisms that underlie this treatment.

### Limitations

One limitation of this study is the method used to evaluate item repetition. In hindsight, it might have been beneficial to include repeated probe items and repeated treatment items to further evaluate how item repetition was related to acquisition. In future studies, we will investigate effects of varying repeated and novel training items as well as probe items.

### Clinical Implications and Future Directions

The results of this study illuminate three clinically relevant findings: (1) The verbal STM component of word processing (activation maintenance) is a potential target for intervention and for some participants, addressing this ability directly by adding a response delay, can improve overall language performance. (2) Item repetition plays a role in improvement potentially through practice effects and/or priming effects. (3) These two variables, item repetition and activation maintenance, may be differentially affected in someone's overall profile of input and output word processing abilities. The results of this study provide greater insight into the nature of the treatment task itself, including its lexical component (words to be repeated) and processing component (the response delay). Both components are important to the success of the therapy, but there is an indication that some may need the temporal processing component of the treatment more than the lexical component.

Future testing is needed to determine how these two components contribute to the success of the treatment and whether those contributions vary depending on the nature of the lexical impairment (semantic and/or phonological), its severity or other factors. Additionally, to better understand the mechanism of this improvement more studies are needed that evaluate the contributions of these two components of lexical processing in different tasks and in the context of various lexical processing profiles (i.e., semantic or phonological input and output impairments). To that end, we are currently investigating the effectiveness of a naming treatment that follows the same principles as the repetition plus response delay treatment (7).

## Conclusion

This study of a treatment for word processing impairment in aphasia focuses on improving one of two parameters of activation that support access to and retrieval of words. The treatment task is repetition and a critical addition to that task is a response delay that for some people with aphasia, challenges their ability to maintain activation of the words that are to be repeated. The results of this study showed that this treatment led to gains in the treatment task (repetition of concrete word pairs after a 5-s delay) for three of our five participants when items in probes were repeated and for one person when the probes used novel items on each probe trial. On the outcome measures, we found evidence indicating that this treatment is specific to those who demonstrate an impairment of activation maintenance in repetition; two participants that demonstrated this deficit made gains on more outcome measures than the other participants in this study.

## Data Availability Statement

The raw data supporting the conclusions of this article will be made available by the authors, without undue reservation.

## Ethics Statement

The studies involving human participants were reviewed and approved by Temple University Institutional Review Board. The patients/participants provided their written informed consent to participate in this study.

## Author Contributions

NM, JO, JS, and RW contributed equally to the content of this research and to the written document. JO and JS provided the treatment and analysis of the data. RW provided statistical analyses and interpretation of the treatment results and outcome measures. NM provided her expertise on theories of lexical processing theories of language and aphasia that postulate a role of verbal STM in language processing. All authors contributed equally to writing as well as to the discussion and interpretation of the results of this study. All authors contributed to the article and approved the submitted version.

## Funding

Research reported in this publication was supported by National Institute on Deafness and other Communication Disorders Center of the National Institutes of Health under award numbers R01DC013196 and R01DC016094.

## Author Disclaimer

The content is solely the responsibility of the authors and does not necessarily represent the official views of the National Institutes of Health.

## Conflict of Interest

The authors declare that the research was conducted in the absence of any commercial or financial relationships that could be construed as a potential conflict of interest.

## Publisher's Note

All claims expressed in this article are solely those of the authors and do not necessarily represent those of their affiliated organizations, or those of the publisher, the editors and the reviewers. Any product that may be evaluated in this article, or claim that may be made by its manufacturer, is not guaranteed or endorsed by the publisher.
