# Supplementary material for: Treatment of the Linguistic and Temporal Components of Lexical Activation to Improve Word Retrieval in Aphasia
Source: Front Rehabil Sci. 2022 Feb 28;3:824684. doi: 10.3389/fresc.2022.824684 (PMC9397957; doi:10.3389/fresc.2022.824684)
Supplement: Supplementary file 1 [file Data_Sheet_1.PDF]

Supplemental Materials

1. Sample treatment screener with Floor = at least 1 pair at 1-second and Ceiling = above 80% at 5-second

Treatment Screener: Adapted from the Psycholinguistic Assessments of Language Processing in Aphasia (PALPA)

5-second delay

Item	Target 1	Target 2	Syllables	Target 1 Response	Target 2 Response	Score
1	window	tractor	4			
2	session	mercy	4			
3	deed	principal	4			
4	letter	gravy	4			
5	cart	hospital	4			
6	feather	hotel	4			
7	moment	valor	4			
8	gravity	length	4			
9	pupil	village	4			
10	folly	member	4			
11	church	potato	4			
12	theory	bonus	4			
13	alcohol	hand	4			
14	summer	onion	4			
15	fact	episode	4			
16	quality	realm	4			
17	wheat	radio	4			
18	miracle	thing	4			
19	treason	purpose	4			
20	night	elephant	4			
Totals				/20	/20	/20

1-second delay

Item	Target 1	Target 2	Syllables	Target 1 Response	Target 2 Response	Score
1	audience	pill	4			
2	wrath	character	4			
3	crisis	tribute	4			
4	battle	monkey	4			
5	attitude	pact	4			
6	clue	opinion	4			
7	elbow	mother	4			
8	effort	dogma	4			
9	tobacco	plane	4			
10	student	funnel	4			
Totals				/10	/10	/10

2. Example of a probe filled in with responses

Target 1		Target 2		Response 1	Response 2	Correct ISO	Correct IAO	Correct String ISO	Correct String IAO
factory	ISO	bison	ISO	factory	bison	2/2	2/2	1/1	1/1
thunder	ISO	coconut	IAO	thunder	/kodəkənət/, coconut	1/2	2/2	0/1	1/1
peroxide	ISO	kennel	ISO	peroxide	kennel	2/2	2/2	1/1	1/1
citizen	ISO	aloe	ISO	citizen	aloe	2/2	2/2	1/1	1/1
termite	ISO	icicle		termite	/aɪskərkəl/	1/2	1/2	0/1	0/1
infant	ISO	gazebo		infant	/kəsibo/	1/2	1/2	0/1	0/1
cereal	ISO	anklet	ISO	cereal	anklet	2/2	2/2	1/1	1/1
duffel		pharmacy	ISO	/təfəl/	pharmacy	1/2	1/2	0/1	0/1
mustache	ISO	kangaroo	ISO	mustache	kangaroo	2/2	2/2	1/1	1/1
oven	ISO	sycamore	ISO	oven	sycamore	2/2	2/2	1/1	1/1
anchovy		pilgrim	ISO	/æntovi/	pilgrim	1/2	1/2	0/1	0/1
chorus	ISO	vanilla	ISO	chorus	vanilla	2/2	2/2	1/1	1/1
recipe	ISO	arcade	ISO	recipe	arcade	2/2	2/2	1/1	1/1
siren	ISO	lavender	ISO	siren	lavender	2/2	2/2	1/1	1/1
daffodil	ISO	tiger	ISO	daffodil	tiger	2/2	2/2	1/1	1/1
appendix		hockey	ISO	/ɛpɛndɪkts/	hockey	1/2	1/2	0/1	0/1
catalog	ISO	sternum	ISO	catalog	sternum	2/2	2/2	1/1	1/1
lentil	ISO	porcelain	ISO	lentil	porcelain	2/2	2/2	1/1	1/1
melon	ISO	detergent		melon	/tətərdʒɪnt/	1/2	1/2	0/1	0/1
alfalfa	ISO	pirate	ISO	alfalfa	pirate	2/2	2/2	1/1	1/1
				**ISO 17/20**	**16/20**	**33/40**	**34/40**	**13/20**	**14/20**
		**Totals**		**IAO 17/20**	**17/20**				
